# A Polyethylenimine-Containing and Transferrin-Conjugated Lipid Nanoparticle System for Antisense Oligonucleotide Delivery to AML

**DOI:** 10.1155/2016/1287128

**Published:** 2016-01-26

**Authors:** Yiming Yuan, Lijing Zhang, Hua Cao, Yi Yang, Yu Zheng, Xiao-juan Yang

**Affiliations:** Department of Geriatric Medicine, State Key Laboratory of Biotherapy, West China Hospital, Sichuan University and Collaborative Innovation Center of Biotherapy, Chengdu 610041, China

## Abstract

Limited success of antisense oligonucleotides (ASO) in clinical anticancer therapy calls for more effective delivery carriers. The goal of this study was to develop a nanoparticle system for delivery of ASO G3139, which targets mRNA of antiapoptotic protein Bcl-2, to acute myeloid leukemia (AML) cells. The synthesized nanoparticle Tf-LPN-G3139 contained a small molecular weight polyethylenimine and two cationic lipids as condensing agents, with transferrin on its surface for selective binding and enhanced cellular uptake. The optimized nitrogen to phosphate (N/P) ratio was 4 to achieve small particle size and high G3139 entrapment efficiency. The Tf-LPN-G3139 exhibited excellent colloidal stability during storage for at least 12 weeks and remained intact for 4 hours in nuclease-containing serum. The cellular uptake results showed extensive internalization of fluorescence-labelled G3139 in MV4-11 cells through Tf-LPN. Following transfection, Tf-LPN-G3139 at 1 *µ*M ASO level induced 54% Bcl-2 downregulation and >20-fold apoptosis compared to no treatment. When evaluated in mice bearing human xenograft AML tumors, Tf-LPN-G3139 suppressed tumor growth by ~60% at the end of treatment period, accompanied by remarkable pharmacological effect of Bcl-2 inhibition in tumor. In conclusion, Tf-LPN-G3139 is a promising nanoparticle system for ASO G3139 delivery to AML and warrants further investigations.

## 1. Introduction

Antisense oligonucleotides (ASO), a type of short single-stranded DNA molecules, theoretically have the potential to reduce cancer gene expression via the complementary binding to a specific target mRNA followed by cleavage or disablement of the mRNA [1, 2]. However, they are limited to clinical applications due to rapid degradation by endo and exo nucleases, weak cellular uptake, and misleading off-target effects [3, 4]. The development of nanoparticle carriers could bypass the above ASO delivery obstacles. Well-designed and formulated nanoparticles, with appropriate size, EPR effect, adjustable surface characteristics, and biocompatibility, could protect ASO from nuclease degradation, increase cellular uptake, and ensure targeted delivery [5–7]. The recently developed examples include polyisohexylcyanoacrylate nanoparticles [8], protamine-oligonucleotide nanoparticles [9], calcium phosphate nanoparticles [10], and transmembrane receptor-targeted lipid nanoparticles [11]. Despite the successful delivery of these ASO nanoparticles* in vitro*, little is known about their* in vivo* efficacy. Moreover, the utility of nanoparticles must be evaluated in a case-by-case basis for each ASO of interest, which is G3139 (oblimersen sodium, Genta, Inc.) in this study. G3139 is an 18-mer phosphorothioate ASO containing unmethylated CpG units. It targets mRNA of Bcl-2, an important antiapoptotic protein [12]. G3139 was launched to clinical phase I study for treating prostate cancer in 2001 [13] and investigated in other types of cancers thereafter [14–18]. The* in vivo* efficacy of G3139 is dependent on its successful delivery to the cytoplasm, whereas it is very difficult to achieve. The objective of this study was to develop a nanoparticle system which is capable of delivering G3139 to acute myeloid leukemia (AML) cells to induce Bcl-2 downregulation and inhibit cancer cell growth. Polyethylenimine (PEI) contains high positive charges, which makes it capable of tightly binding to negatively charged nucleic acids. It also owes the endosomolytic activity, benefiting cellular uptake and ASO intracellular release [19, 20]. In this study, a low molecular weight PEI, PEI1200, was selected for the formulation because of less cytotoxicity than the popular transfection agent PEI 25000 [21–23]. A ligand transferrin (Tf) was conjugated to nanoparticle surface for the purpose of targeted delivery to AML cells as most malignant cells overexpress transferrin receptor (TfR) [24]. The formulated nanoparticle Tf-LPN-G3139 with optimized nitrogen to phosphate (N/P) ratio was characterized for stability, cellular uptake in AML cell line MV4-11,* in vitro* pharmacology, and* in vivo* anticancer activity in MV4-11 tumor-bearing mouse model.

## 2. Materials and Methods

### 2.1. Materials

Cholesterol (Chol), didecyldimethylammonium bromide (DDAB), polyethylenimine 1200 (PEI1200, MW 1200), d-*α*-tocopherol polyethylene glycol succinate (TPGS), human holo-transferrin (Tf), 2-iminothiolane (Traut's reagent), and other chemicals and reagents were purchased from Sigma-Aldrich Chemical Co. (St. Louis, MO). 1,2-Dioleoyl-3-trimethylammonium-propane (DOTAP) and distearoyl phosphatidylethanolamine-N-[maleimide-polyethylene glycol, MW 2000] (Mal-PEG2000-DSPE) were purchased from Avanti Polar Lipids (Alabaster, AL). All tissue culture media and supplies were purchased from Invitrogen (Carlsbad, CA). Antisense oligonucleotides used in this study were fully phosphorothioated. These include G3139 (5′-TCT CCC AGC GTG CGC CAT-3′) and fluorescein-labeled G3139, FAM-G3139, which were custom-synthesized by TsingKe (Chengdu, China).

### 2.2. Cell Culture

Acute myeloid leukemia cell line MV4-11 was cultured in RPMI 1640 media supplemented with 10% heat-inactivated fetal bovine serum (FBS) (Invitrogen), 100 U/mL penicillin, 100 *μ*g/mL streptomycin, and L-glutamine at 37°C in a humidified atmosphere containing 5% CO_2_.

### 2.3. Synthesis of Tf-LPN-G3139

A modified ethanol dilution method [25] was used to synthesize LPN-G3139. A lipid mixture of DOTAP/DDAB/Chol/TPGS at a molar ratio of 30/30/39/1 was dissolved in ethanol and quickly injected into an aqueous solution containing PEI1200. The above micelle mixture was then quickly injected into G3139 dissolved in HEPES-buffered saline (HBS, composed of 20 mM HEPES, 145 mM NaCl, pH 7.4) at a ratio lipid : G3139 of 3 : 1 (w/w) and nitrogen to phosphate (N/P) ratios of 2 to 8, adjusted by content of PEI1200 in micelles. The sonication was then applied to get the nanoparticle with 50 *μ*g/mL G3139 in 20% ethanol, designated as LPN-G3139. The ethanol in the suspension was removed by dialysis against HBS using a MWCO 10,000 Dalton Float-A-Lyzer.

A postinsertion method [26] was adopted to incorporate Tf ligand into LPN-G3139 or control nanoparticle. Holo (diferric) Tf in HEPES-buffered saline (HBS, pH 8, containing 5 mM EDTA) reacted with 5x Traut's reagent to yield holo-Tf-SH. Free Traut's reagent was removed by dialysis using a MWCO 10,000 Dalton Float-A-Lyzer and against HBS. Holo-Tf-SH was coupled to micelles of Mal-PEG2000-DSPE at a protein-to-lipid molar ratio of 1 : 10. The resulting Tf-PEG2000-DSPE micelles were then incubated with the LPN-G3139 for 1 hour at 37°C at Tf-PEG2000-DSPE to DOTAP/DDAB/Chol/TPGS lipids molar ratio of 1 : 100. The above formulated nanoparticle was designated as Tf-LPN-G3139.

For synthesizing, fluorescence-labeled nanoparticles G3139 were spiked with 10% FAM-G3139. As controls, the nanoparticle without PEI1200, named as LN-G3139, and the empty vector Tf-LPN were synthesized in parallel.

### 2.4. Particle Size and G3139 Entrapment Efficiency

The particle sizes of the nanoparticles were analyzed on a NICOMP Particle Sizer Model 370 (Particle Sizing Systems, Santa Barbara, CA). A volume-weighted Gaussian distribution analysis was used to determine the mean particle diameter. All measurements were carried out in triplicate.

G3139 concentration was determined by dissolving LPN-G3139 using 0.5% SDS followed by the fluorometry to determine fluorescence derived from FAM-G3139 using the excitation at 488 nm and emission at 520 nm and against a standard curve of fluorescence intensity versus G3139 concentration. Loading efficiency of G3139 in the LPNs was calculated based on the ratio of G3139 concentration in particles before and after dialysis.

### 2.5. Colloidal and Serum Stability

The colloidal stability of Tf-LPN-G3139 was evaluated by monitoring changes in the mean particle diameter during storage at 4°C. To evaluate the ability of the Tf-LPN-G3139 to retain G3139 and protect it against nuclease degradation, the formulation was mixed with FBS at a 1 : 4 (v/v) ratio and incubated at 37°C. At various time points, aliquots of each sample were loaded onto a 3% low melting point agarose gel. Electrophoresis was performed and the G3139 bands were visualized by SYBR Gold (Invitrogen) staining. The densities of G3139 band were measured and analyzed by the ImageJ software (NIH Image, Bethesda, MD).

### 2.6. Cellular Uptake

Cellular uptake of Tf-LPN-G3139, loaded with G3139 spiked with 10% fluorescent FAM-G3139, or controls, was evaluated in AML cell MV4-11* in vitro*. For the studies, 4 × 10^5^ cells were incubated at 37°C with 1 *μ*M G3139 entrapped in Tf-LPN-G3139. After 1-hour incubation, the cells were washed with PBS for three times and observed on a Nikon fluorescence microscope (Nikon, Küsnacht, Switzerland).

### 2.7. Transfection Studies

Leukemia cells were plated in 6-well tissue culture plates at 1 × 10^6^/well in RPMI1640 medium containing 10% FBS. An appropriate amount of Tf-LPN-G3139, or control formulations, was added into each well to yield a final G3139 concentration of 1 *μ*M. After a 4-hour incubation at 37°C in a CO_2_ incubator, the cells were transferred to a fresh medium, incubated for another 48 hours, and then harvested for analyzing Bcl-2 protein level and apoptosis.

### 2.8. Western Blot

To analyze Bcl-2 protein level, Western blot was carried out as described previously [27] using monoclonal murine anti-human Bcl-2 antibody (Dako, Carpinteria, CA). Bcl-2 protein expression levels were quantified by ImageJ software and normalized to GAPDH level from the same sample.

### 2.9. Analysis of Apoptosis by Caspase Activation

To analyze cellular apoptosis, caspase-8 and caspase-9 activities were measured on untreated and Tf-LPN-G3139-treated cells using the Caspase-Glo Assay Systems (Promega). Briefly, 5 × 10^3^ MV4-11 cells were plated in a white-walled 96-well plate. After transfection, a luminogenic caspase-8 or caspase-9 substrate, was added with a 1 : 1 ratio of reagent to cell solution. After 90 minutes at room temperature, the substrate was cleaved by activated caspase and the intensity of a luminescent signal was measured by a Fluoroskan Ascent FL luminometer (Thermo Electron Corp.). Differences in caspase activities in Tf-LPN-G3139-treated cells compared with untreated cells were expressed as fold-change in luminescence.

### 2.10. Evaluation of Antitumor Activity

MV4-11 cells (5 × 10^6^) were subcutaneously inoculated into the flank of the female athymic mice. Palpable tumors developed within 4-5 days after inoculation. From day 5 of postinoculation, the tumor-bearing mice were injected i.v. with PBS (pH 7.4), free G3139, or Tf-LPN-G3139 once daily at dose of 10 mg/kg. Empty Tf-LPN without G3139 was also included as a control treatment. Five mice were used in each treatment group. Antitumor activity was determined by measuring the tumor size (width and length) using a Vernier caliper at a series of time points. Tumor volume was calculated by the formula tumor volume = 1/2 × length (mm) × (width (mm))^2^. Mice were sacrificed once the tumor size reached 1,500 mm^3^.

### 2.11. Immunohistochemical Analysis of Bcl-2 Expression in Tumor

Immunohistochemical stain of Bcl-2 and H&E counterstain were performed as previously reported [28] after fixing tumor samples from mice treated by Tf-LPN-G3139 or control formulations. Slides with H&E staining only were used as control.

### 2.12. Statistical Analysis

Data was generally represented as mean ± standard deviations (SD). Comparisons between groups were made by 2-tailed Student's *t*-test. *p* value < 0.05 was used as the cutoff for defining statistically significant differences.

## 3. Results

### 3.1. Tf-LPN-G3139 Components, Particle Size, and Entrapment Efficiency

The formulated nanoparticle Tf-LPN-G3139 was composed of ASO G3139, PEI1200, and lipids DOTAP/DDAB/Chol/TPGS (molar ratio 30/30/39/1) at a lipid : G3139 weight ratio of 3 : 1. The N/P ratio of the formulation was optimized by adjusting PEI1200 amount in the system and screened by particle size and G3139 entrapment efficiency. As shown in Table 1, with increased N/P ratio from 2 to 8, nanoparticle size reduced from approximately 229 nm to 133 nm, while G3139 entrapment efficiency increased by approximately 18%. When PEI1200 was completely removed from the nanoparticle, the diameter of the particle, Tf-LN-G3139, increased to 341 nm and the G3139 content decreased to 70%, which verified the importance of PEI1200 in entrapping ASO and condensing nanoparticles. LPN-G3139 without transferrin ligand had the comparable particle size (~162 nm) to Tf-LPN-G3139 (~169 nm) (*p* value = 0.131). Among the three nanoparticle formulations which exhibited <200 nm sizes and high G3139 entrapment efficiencies (~95%), Tf-LPN-G3139 with N/P ratio 4 had the least positive charges. Considering that cationic particles tend to bind serum protein (such as albumin) in systemic circulation, which lead to opsonization and clearance by the reticuloendothelial system (RES), Tf-LPN-G3139 with N/P ratio 4 was therefore selected for the next step characterization of stability and pharmacological activities.

### 3.2. Stabilities of Tf-LPN-G3139

**Figure 1:**
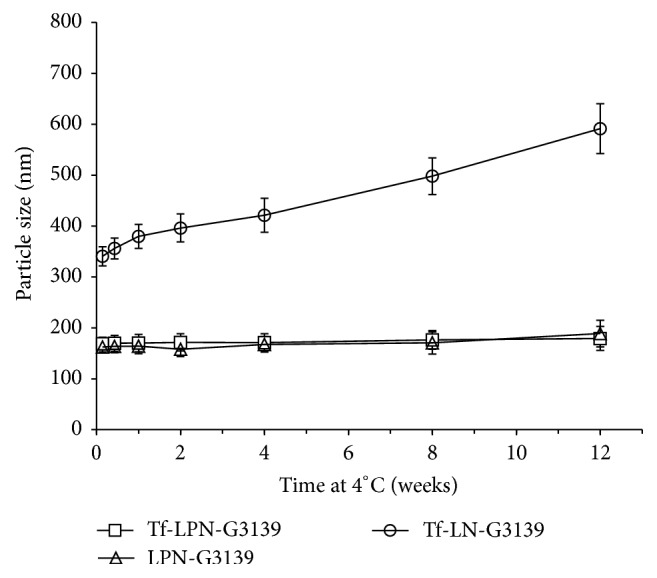
Colloidal stability over storage at 4°C. The values in the plot represent the mean particle sizes of 3 measurements. Error bars were standard deviations, *n* = 3.

As a colloidal delivery system, nanoparticle Tf-LPN-G3139 must remain stable for relatively long periods before it is utilized. When stored in at 4°C, Tf-LPN-G3139 remained stable for at least 12 weeks with no significant change in mean diameter (*p* values > 0.093) (Figure 1). LPN-G3139 without transferrin had the similar profile of particle size change over storage time compared to that of Tf-LPN-G3139, while particle size of control nanoparticle without PEI1200 (Tf-LN-G3139) at week 12 was approximately 1.2-fold of that upon production.

**Figure 2:**
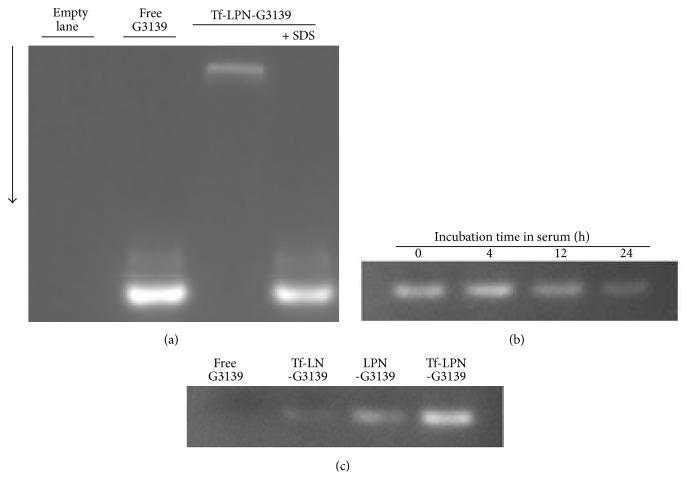
Serum stability. The electrophoresis bands in the figure represent retained intact G3139 in serum after incubation. (a) Release of G3139 from Tf-LPN-G3139 by SDS in the absence of serum. (b) Time course of Tf-LPN-G3139 incubation in serum. (c) Tf-LPN-G3139 and control formulations after 4-hour incubation in serum.

Estimating the intactness of ASO in biological environments is critical to clarify the fate of ASO loaded nanoparticles after administration. The stability of Tf-LPN-G3139 in serum was hence assessed by electrophoresis following incubation of Tf-LPN-G3139 in FBS. In the absence of serum, Tf-LPN-G3139 was retained in nanoparticle without release except for the dissolution by SDS (Figure 2(a)). When incubated in serum, Tf-LPN was able to protect G3139 from serum nuclease degradation. Although the amount of intact G3139 retained in the particle decreased over time, there was still 99% G3139 in Tf-LPN after 4-hour incubation (Figure 2(b)). In contrast, free G3139 completely disappeared at the same timepoint. The Tf-LN and LPN retained only approximately 1/8 and 1/3 of the starting amount (Figure 2(c)). These results indicated that both PEI1200 and transferrin in the nanoparticle Tf-LPN were critical to keep G3139 intact and elongate the elimination half-life in systemic circulation.

### 3.3. Cellular Uptake of G3139 Delivered by Tf-LPN-G3139

**Figure 3:**
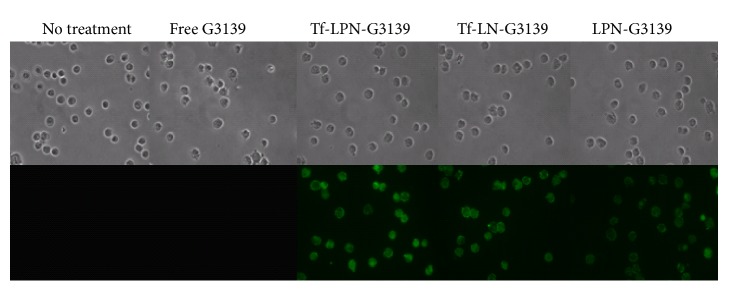
Cellular uptake. Internalization of G3139 spiked with 1/10 G4243 in MV4-11 cells via Tf-LPN-G3139 or control nanoparticles, visualized under a fluorescence microscope.

The delivery efficiency of Tf-LPN-G3139 was firstly evaluated by cellular uptake of G3139. After incubating AML MV4-11 cells with fluorescence-labeled Tf-LPN-G3139 or control formulations for 1 hour, the cells were observed under fluorescence microscope. As shown in Figure 3, there was little G3139 uptake in free G3139-treated cells. The Tf-LPN delivered significantly higher level of G3139 to MV4-11 cells compared to the naked G3139 and two control formulations. The difference in cellular uptaken levels between Tf-LPN-G3139 and Tf-LN-G3139 without PEI1200 was similar to that between Tf-LPN-G3139 and the nanoparticle without transferrin (LPN-G3139), implying that both transferrin and PEI1200 contributed significantly to G3139 cellular uptake, which should be thorough TfR directed endocytosis [23] and endosomolytic activity [19], respectively.

### 3.4. Downregulation of Bcl-2 Expression and Induction Apoptosis by Tf-LPN-G3139

**Figure 4:**
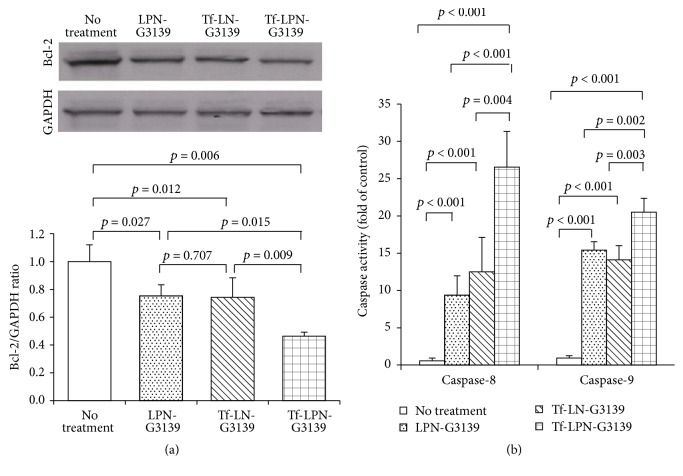
*In vitro* pharmacology. (a) Bcl-2 protein expression by Western bot. (b) Caspase activities for measuring apoptosis. The values in the plots represent the means of 4 separate experiments. Error bars were standard deviations, *n* = 4.

Since the anticancer mechanism of G3139 is to downregulate expression of antiapoptotic protein Bcl-2, the delivery efficiency of Tf-LPN-G3139 was then estimated by Western blot for measuring cellular Bcl-2 protein level. As shown in Figure 4(a), after transfection of MV4-11 cells with Tf-LPN-G3139 there was 54% Bcl-2 downregulation. The non-PEI1200 formulation Tf-LN-G3139 induced 26% Bcl-2 downregulation, which was comparable to the inhibition by LPN-G3139 (24%). Having demonstrated the pharmacological effect of reducing Bcl-2 expression, Tf-LPN-G3139 transfected cells were then assessed for apoptosis by quantifying caspase-8 and caspase-9 activities. As shown in Figure 4(b), following Tf-LPN-G3139 treatment, cellular caspase-8 and caspase-9 activities were approximately 27- and 21-fold of untreated cells, indicating remarkably enhanced apoptosis by Tf-LPN-G3139. The control nanoparticles LPN-G3139 and Tf-LN-G3139 triggered 45% and 49% less apoptosis, respectively, consistent with their performance in Bcl-2 inhibition.

### 3.5. Tumor Growth Suppression by Tf-LPN-G3139

**Figure 5:**
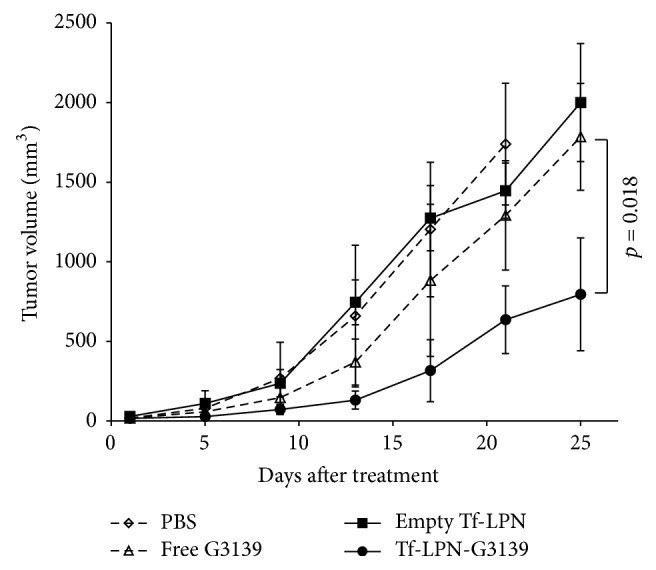
Tumor suppression. The values in the plots represent the mean tumor volume from five tumor-bearing mice receiving the same treatment. Error bars were standard deviations, *n* = 5.

*In vivo* anticancer activity of Tf-LPN-G3139 was estimated in mice bearing AML xenograft tumors. The tumor model was established in nude mice by subcutaneous implantation with MV4-11 cells. Tumors with ~50 mm^3^ volumes were developed within 5 days, which reached a size of >1,500 mm^3^ in 25 days in the absence of G3139 treatment (PBS only). For the therapeutic study, the mice were i.v. injected with Tf-LPN-G3139 once daily starting from day 5 after inoculation. The control group mice were given naked G3139 and the empty nanoparticle instead. Tumor growth in mice treated with Tf-LPN-G3139 was inhibited by 61% (*p* value < 0.02) (Figure 5) and life span increased by 1-fold at the end of the treatment period. In contrast, neither free G3139 nor empty nanoparticle Tf-LPN had significant effects on inhibiting tumor growth. Although it was observed that free G3139 induced mild tumor suppression over the first two weeks of treatment, the impact did not last thereafter till the end of the treatment.

### 3.6. Bcl-2 Expression in AML Xenograft Tumor

**Figure 6:**
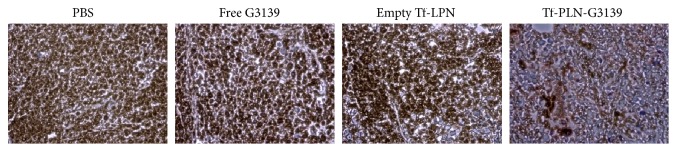
Immunohistochemical staining of tumor Bcl-2.

The antitumor activity of G3139 has been attributed to both downregulation of Bcl-2 expression [29, 30] and its immunomodulatory effects [31]. To investigate if the observed tumor suppression by Tf-LPN-G3139 was caused by G3139 antisense effect, Bcl-2 expression in tumor tissues was measured by immunohistochemical staining following multiple doses of Tf-LPN-G3139 or controls. As shown in Figure 6, Bcl-2 expression (stained in brown color) in Tf-LPN-G3139-treated mice tumors was significantly downregulated, compared to ineffectiveness of PBS, free G3139, and empty vector. Considering that free G3139 induced mild tumor suppression in the first two weeks of treatment without corresponding Bcl-2 downregulation, the slight antitumor effect of free G3139 was probably due to immune response caused by C_p_G islands in G3139.

## 4. Discussion

The objective of this study was to develop an efficient nanoparticle system for delivery of G3139, an 18-mer ASO targeting the mRNA of antiapoptotic protein Bcl-2, to AML cells. The basic compositions of the developed nanoparticle vector, designated as Tf-LPN, included cationic lipids DOTAP and DDAB, TPGS, PEI1200, and TfR-targeting ligand transferrin. The reasons for selecting PEI1200 and transferrin have been stated in Introduction. The control formulations without PEI1200 (Tf-LN) and without transferrin (LPN) were designed and included in the series of assessments to verify the necessity to adopt these two components in the case of G3139. The results from the study have confirmed the importance of PEI1200 in binding ASO (Table 1) and stabilizing nanoparticle over storage (Figure 1), which could be explained by its high density of positive charges condensing ASO into cationic lipid particles. The nanoparticle formulation Tf-LN-G3139 showed less colloidal stability with the more increases in particle size over storage (Figure 1), compared to Tf-LPN-G3139 and LPN-G3139, because of the lack of PEI condensation. The critical role of PEI1200 in cellular uptake (Figure 3) could be contributed to its endosomolytic activity [19, 20]. Transferrin was shown as critical as PEI1200 in G3139 cellular uptake, which could attribute to TfR-directed endocytosis [24]. Bicationic lipid concept was introduced in G3139 nanoparticle design of this study to avoid cytotoxicity caused by a high percentage of single cationic lipid component (data not shown). TPGS is a water-soluble derivative of vitamin E and has hydrophile-lipophile balance of 13. Its bulky structure and large surface area characteristics make it an excellent emulsifier and matrix material. Moreover, it has been found that coadministration of TPGS could reduce cytotoxicity, inhibit P-glycoprotein mediated multidrug resistance, enhance delivery, and increase the oral bioavailability of anticancer drugs [32, 33].

Tf-LPN-G3139 successfully delivered ASO G3139 to AML cells MV4-11* in vitro*, as evidenced by extensive G3139 cellular uptake, over 50% inhibition of Bcl-2 expression and induction of more than 20-fold of caspases-based apoptosis (Figure 4). When estimated in mice bearing AML xenograft tumor, Tf-LPN-G3139 suppressed tumor growth by approximately 60% at the end of the treatment period (Figure 5), which was mostly due to the antisense mechanism of G3139 to target Bcl-2 expression, as shown by the Bcl-2 immunohistochemical staining results (Figure 6). The contribution of lipid and polymer components to anticancer activity was denied because empty vector Tf-LPN showed no efficacy in AML tumor suppression. Interestingly, it was found that free G3139 triggered mild tumor suppression during the first two weeks of treatment, which could not be explained by its antisense mechanism since no corresponding Bcl-2 reduction was observed in free G3139-treated tumors (Figure 6). It has been previously established that ASO G3139 may function by both downregulating Bcl-2 expression to induce cellular apoptosis and by activating the immune cells such as dendritic cells (DC), macrophages, and natural killer (NK) cells, through its 2 CpG motifs and toll-like receptor 9 (TLR9) activation [29–31]. Therefore, the mild tumor growth inhibition by free G3139 was probably due to G3139 CpG immune stimulation instead of antisense binding to Bcl-2 mRNA. Nevertheless, free G3139's anticancer effect observed in this study was just temporary. The consistent and long-term effect shown by Tf-LPN-G3139 is still what should be pursued and warrants further investigations.

## 5. Conclusion

In this study a novel PEI-containing and Tf-conjugated nanoparticle system Tf-LPN was developed for ASO G3139 delivery to AML. It exhibited small particle size (169 nm), high G3139 loading (95%), and excellent stability over storage and in serum. When estimated* in vitro* and in tumor-bearing mice, Tf-LPN-G3139 induced 54% downregulation of target gene expression and 61% tumor suppression. These results showed that Tf-LPN-G3139 is a promising nanoparticle system for ASO G3139 delivery to AML and warrants further investigations.

## Figures and Tables

**Table 1:** Particle size distribution and G3139 entrapment efficiency of Tf-LPN-G3139 and control nanoparticles (mean ± SD; *n* = 5).

Nanoparticle	N/P ratio	Size (nm)	G3139 entrapment efficiency (%)
Tf-LPN-G3139	8	133.4 ± 7.6	95.8 ± 2.9
Tf-LPN-G3139	6	158.3 ± 9.8	94.4 ± 3.1
Tf-LPN-G3139	4	169.2 ± 12.5	95.0 ± 4.2
Tf-LPN-G3139	2	229.2 ± 10.5	78.4 ± 3.7
Tf-LN-G3139	1	340.7 ± 18.9	70.1 ± 4.2
LPN-G3139	4	162.4 ± 9.7	93.2 ± 3.4
Tf-LPN	—	12.4 ± 1.3	—
